# Identification, Characterization, and Modeling of a Bioinsecticide Protein Isolated from Scorpion Venom gland: A Three-Finger Protein

**DOI:** 10.61186/ibj.3885

**Published:** 2023-04-26

**Authors:** Masoumeh Baradaran, Masoud Mahdavinia, Maryam Naderi Soorki, Sahand Jorfi

**Affiliations:** 1 Toxicology Research Center, Medical Basic Sciences Research Institute, Ahvaz Jundishapur University of Medical Sciences, Ahvaz, Iran;; 2 Department of Toxicology, School of Pharmacy, Toxicology Research Center, Medical Basic Sciences Research Institute, Ahvaz Jundishapur University of Medical Sciences, Ahvaz, Iran;; 3 Department of Biology, Faculty of Science, Shahid Chamran University of Ahvaz, Ahvaz, Iran;; 4 Environmental Technologies Research Center, Ahvaz Jundishapur University of Medical Sciences, Ahvaz, Iran

**Keywords:** Peptides, Scorpion venoms, Three-finger toxin

## Abstract

ABSTRACT

**Background:**

The majority of insecticides target sodium channels. The increasing emergence of resistance to the current insecticides has persuaded researchers to search for alternative compounds. Scorpion venom gland as a reservoir of peptides or proteins, which selectively target insect sodium channels. These proteins would be an appropriate source for finding new suitable anti-insect components.

**Methods:**

Transcriptome of venom gland of scorpion *M. eupeus* was obtained by RNA extraction and cDNA library synthesis. The obtained transcriptome was blasted against protein databases to find insect toxins against sodium channel based on the statistically significant similarity in sequence. Physicochemical properties of the identified protein were calculated using bioinformatics software. The 3D structure of this protein was determined using homology modeling, and the final structure was assessed by MD simulation.

**Results:**

The sodium channel blocker found in the transcriptome of *M. eupeus* venom gland was submitted to the GenBank under the name of meuNa10, a stable hydrophilic protein consisting of 69 amino acids, with the molecular weight of 7721.77 g/mol and pI of 8.7. The tertiary structure of meuNa10 revealed a conserved CS-alpha/beta domain stabilized by eight cysteine residues. The meuNa10 is a member of the 3FP superfamily consisting of three finger-like beta strands.

**Conclusion:**

This study identified meuNa10 as a small insect sodium channel-interacting protein with some physicochemical properties, including stability and water-solubility, which make it a good candidate for further in vivo and in vitro experiments in order to develop a new bioinsecticide.

## INTRODUCTION

Scorpion venom is a rich source of biologically active molecules, including peptides, proteins, enzymes, amines, and nucleotides^[^^1^^]^. Toxic peptides and proteins (toxins) in the scorpion venom gland can modify the function of the ion channels (sodium, potassium, chloride, and calcium)^[^^2^^,^^3^^]^.

Na_v_s are integral transmembrane proteins that are widely distributed on the cell membranes of both invertebrates and vertebrates^[^^4^^]^ and play a vital role in the depolarizing phase of the action potential in most excitable cells^[^^5^^]^. Due to crucial roles of Na_v_s in membrane excitability, these proteins are targeted by many plants or animals neurotoxins for defense or predation^[^^6^^]^. Na_v_s are also a great target for some insecticides, including DDT, oxadiazines^[^^7^^]^, and synthetic pyrethroids^[^^8^^]^; however, some insect species are resistant to these pesticides. Considering the emergence of the increasing resistance to the conventional insecticides, it is important to find new natural components as alternative insecticides with less side effects^[^^4^^,^^9^^]^.

Scorpion toxins specific for insect Na_v_s have been considered as one of the most promising options for insecticide^[^^10^^]^. Some scorpion toxins influence the sodium channels of insects but have no effect on mammalian sodium channels^[^^3^^]^. Hence, they are appropriate candidates for anti-insect assay. Although the binding sites of different toxins on the sodium channels are variable, in some cases, simultaneous use of two toxins causes allosteric effects and increases the lethal effects of the insecticides. For instance, the affinity of the toxin batrachotoxin to Na_v_s enhances in the presence of pyrethroid and DDT^[^^11^^]^. The effect of pyrethroid has also been reported to be amplified up to 100 times when using with neurotoxins such as toxin II, a sea anemone toxin, and brevetoxin^[^^12^^]^. 

Blockers or modifiers of insect sodium channels originated from scorpion venom are attractive candidates for the production and development of novel insecticides^[^^13^^]^. Given the importance of identifying new bioinsecticide, in this study, the transcriptome of the venom gland of *M. eupeus* was analyzed to find a new powerful insect toxin with an action on the insect Na_v_s. After identifying the potent protein, its physicochemical characteristics and 3D structure were determined and discussed.

## MATERIALS AND METHODS

**Sample preparation, RNA extraction, and cDNA library synthesis**

Scorpion samples of *M. eupeus* were collected from deserts of Khuzestan Province, Ahvaz, Iran. The authenticity of the species was confirmed in the Laboratory of Toxicology Research Center, Ahvaz Jundishapur University of Medical Sciences. To extract RNA, venom glands of the confirmed scorpions were separated and collected in a Petri dish. RNA extraction and cDNA library synthesis were performed as reported previously^[^^14^^]^. cDNA sequences were obtained from Sanger sequencing and analyzed by ORF finder (https://www.ncbi.nlm.nih.gov/orffinder/) to find ORFs in non-redundant transcripts of scorpion venom proteins and peptides.

**Transcriptome analysis of the** ***M. eupeus*** **venom gland**

Transcriptome of the venom gland of *M. eupeus* was blasted against Uniprot (https://www.uniprot.org/blast) and NCBI (https://blast.ncbi.nlm.nih.gov/Blast.cgi) using Blastx and Blastp to identify a protein with the greatest similarity to anti-insect sodium channel toxins that previously identified from scorpion venom and its closely related species, including mite, spider, tick, termite, ant, fly, and wasp. The transcript with the highest identity (E-value < 10^-3^) to anti-insect sodium channel blockers in the protein databases was preserved and subjected to further analysis.

**Physicochemical properties and 3D structure determination of anti-insect protein**

The amino acid sequence alignment of the new identified protein with its similar sequences was created using the MUSCLE tool in MEGA11 software^[^^15^^]^. SignalP-6.0 (https://services.healthtech.dtu.dk/service. php?SignalP) was used to predict the potent signal peptide. Molecular weight, theoretical pI, half-life, instability index, GRAVY, and aliphatic index of the identified protein were determined by Protparam online server (https://web.expasy.org/protparam/). Water solubility of the protein was determined using the peptide property calculator online tool INNOVAGEN (http://www.innovagen.com/proteomics -tools). Conserved domains of the discovered protein were determined by searching the protein sequences against MOTIF search (https://www.genome.jp/tools/ motif/). The 3D structure of the protein was determined via homology modeling using three online servers: 

I-TASSER (https://zhanggroup.org/I-TASSER/), SWISS-MODEL (https://swissmodel. expasy.org/), and PHYRE2 (http://www.sbg.bio.ic.ac.uk/~phyre2/ html/page.cgi?id=index). In order to evaluate the energy profile of the predicted structure, ProSA web server (https://prosa.services.came.sbg.ac.at/prosa.php) was utilized. This server uses Z-score for calculating the overall quality and measuring the deviation of total energy for the predicted protein structures^[^^16^^]^. More negative Z-score represents more valid structure^[^^17^^]^. A structural alignment was performed using Pymol molecular visualization tool (www.pymol.org) to compare structures obtained from different servers. Finally, the selected structure with more negative Z-score was further refined by MD simulation using GROMACS package (v. 2021)^[^^18^^]^ at a time step of 5 fs for 100 ns (50 million steps). MD simulation was carried out at constant temperature (310 K) and pressure (1 atm). The MD output trajectories were analyzed to calculate RMSD to determine the stability of the structure, RMSF to assess the flexibility of residues, Rg to evaluate compactness and stability, SASA to measure exposure of the new identified protein to the solvent and examine the secondary structure^[^^19^^]^. The maintenance of the secondary structure during MD simulation was analyzed using the DSSP program (https://swift.cmbi. umcn.nl/gv/dssp/). Visualization analysis of all 3D structures and structural alignments were performed using Pymol.

**Fig. 1:**
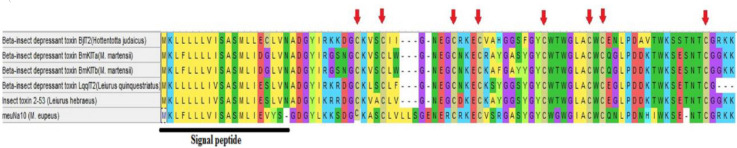
Amino acid sequence alignment of meuNa10 with the most similar scorpion proteins: BjT2 (GenBank ID: P24336), BmKITa (GenBank ID: Q9XY87), BmKITb (GenBank ID: Q95WX6), LqqIT2 (GenBank ID: P19855), Insect toxin 2-53 (GenBank ID: P68726). Signal peptides are represented with the thick black line. Red arrows indicate the conserved cysteine residues.

## RESULTS

**Transcriptome analysis of** ***M. eupeus*** **and identification of the anti-insect protein**

Transcriptome analysis of *M. eupeus* using BLAST against the currently identified proteins deposited in Uniprot and NCBI databases revealed a potent anti-insect sodium channel blocker protein, which named meuNa10 and deposited in the GenBank under the accession number of KU316194. A multiple sequence alignment containing the amino acids of meuNa10 and similar proteins was generated (Fig. 1). All the proteins similar to the meuNa10 were beta-insect depressant toxin collected from the venom of scorpion species. The name of all proteins and their accession numbers are indicated in the Figure 1.

**Physicochemical properties and characterization of meuNa10**

**Fig. 2:**
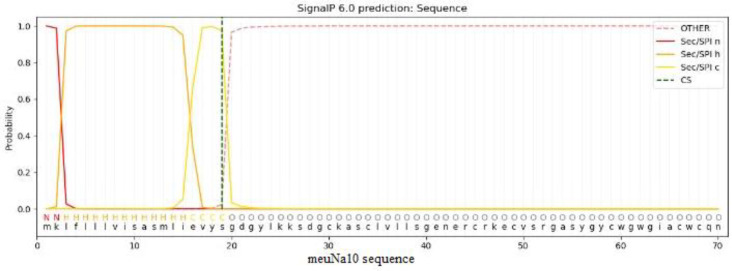
Cleavage site of the signal peptide of meuNa10. Green dashed line shows the cleavage site.

**Fig. 3:**
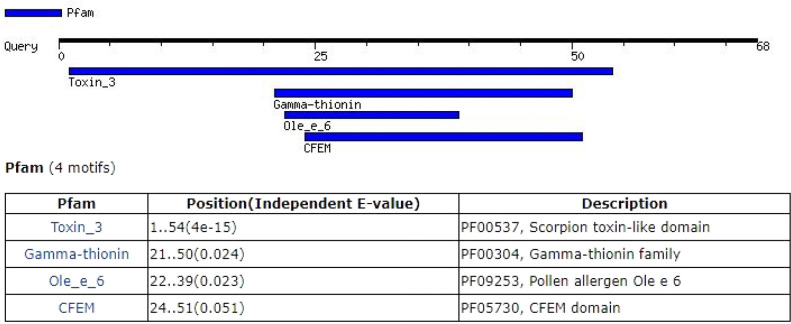
Conserved domains in the structure of meuNa10.

A 468-nucleotides cDNA encoded meuNa10. A signal peptide with a probability more than 0.9991 was predicted for the meuNa10 (Table 1). The cleavage site of the signal peptide was between amino acids numbers 19 and 20 (Fig. 2). Accordingly, meuNa10 was composed of a 19-amino acid signal peptide and a 69-amino acid mature protein. The mature protein of meuNa10 was a water-soluble protein with a molecular weight of 7721.77 g/mol and theoretical pI of 8.7. The half-life of 30 hours in mammalian reticulocytes, >20 hours in yeast, and >10 hours in *Escherichia coli *were estimated for meuNa10. Due to the instability index of 34.10 that calculated for the meuNa10, it was considered as a stable protein. An aliphatic index of 52.32 and a GRAVY of -0.69 were also determined for the meuNa10. Searching for the conserved domains revealed that the meuNa10 included of a main domain (Toxin_3) and three subdomains (Gamma_thionin, Ole_e_6, and CFEM) inside the main domain (Fig. 3).

**Table 1:** Parameters related to signal peptide prediction

**Protein type**	**Signal peptide** **(Sec/SPI)**	**Lipoprotein signal peptide**	**TAT signal peptide (Tat/SPI)**	**TAT Lipoprotein signal peptide (Tat/SPII)**	**Pilin-like signal peptide (Sec/SPIII)**
likelihood	0.9991	0.0002	0.0002	0.0002	0.0002

**Three-dimensional structure determination and MD simulation of meuNa10**

**Fig. 4:**
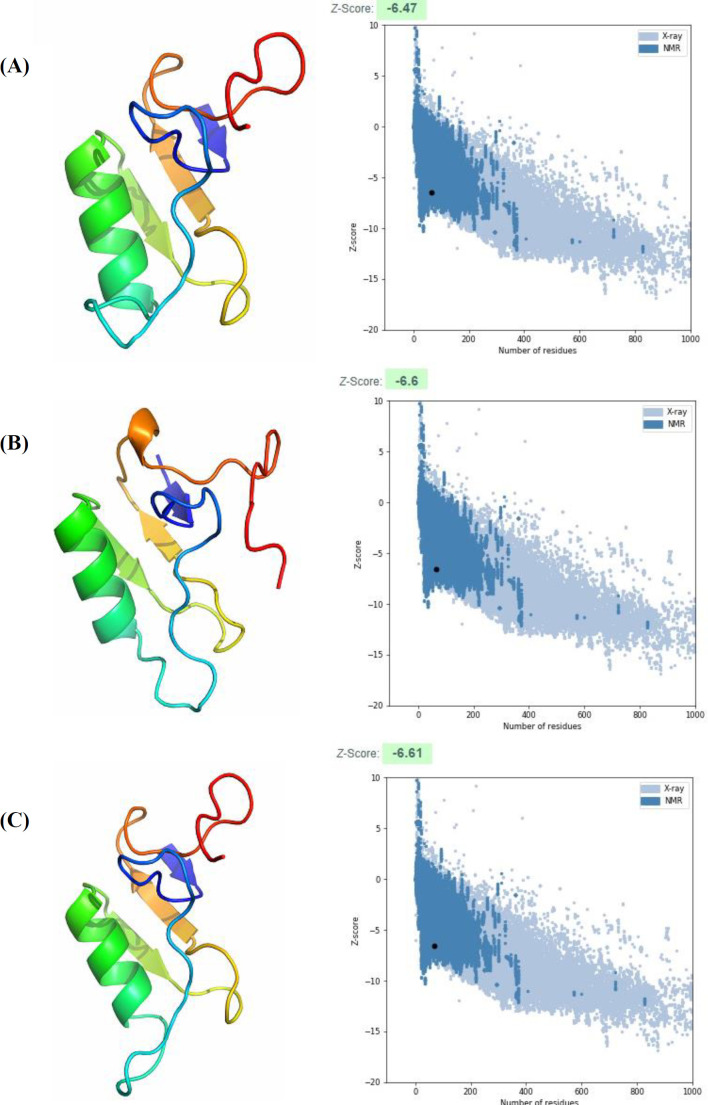
Three-dimensional structure generated for meuNa10 using three different servers, including (A) PHYRE-2, (B) I-TASSER, and (C) SWISS-MODEL. Plots related to model validation using the Z-score calculation are given on the right. The black dots in the plot show the location of the predicted structures. Z-score of meuNa10 models obtained from all three servers are between -10 and 10. Protein structures were visualized and designed using PyMOL tool.

**Fig. 5:**
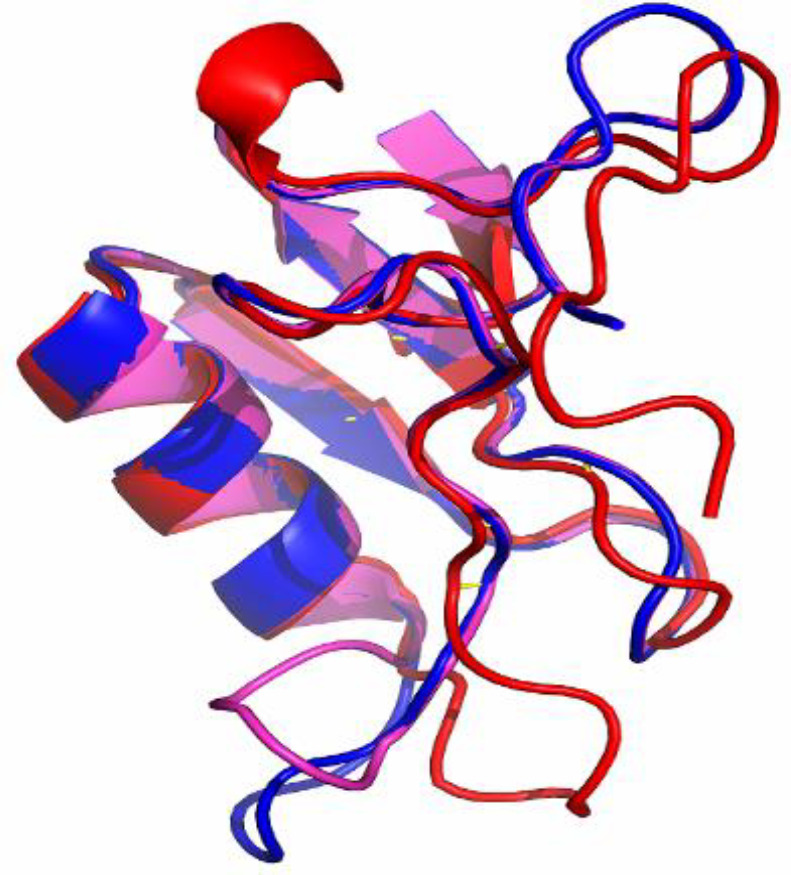
Structural alignment of three models of meuNa10 from three different servers. The red, pink, and blue structures were obtained from I-TASSER, PHYRE-2, and SWISS-MODEL, respectively.

**Fig. 6:**
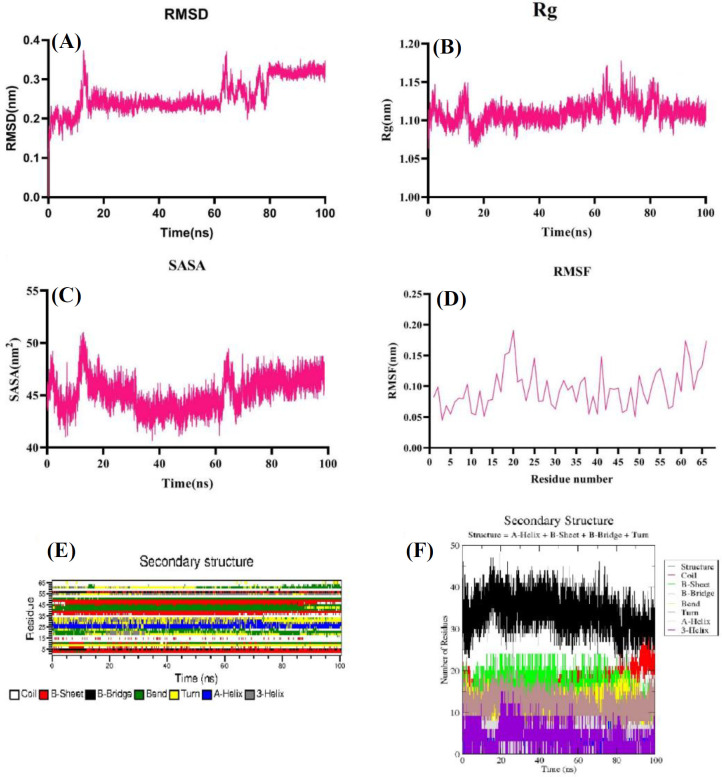
MD simulation analysis of meuNa10 model during 100 ns. (A) RMSD plot of backbone Cα atoms of meuNa10, showing the steady state of the model from 20 to 60 ns and after 80 ns; (B) Rg plot, indicating stability and compactness of meuNa10 from 20 to 60 ns and after 80 ns; (C) SASA plot, representing solvent accessible area around 45 nm^2^ in most of the time during the simulation; (D) RMSF plot, revealing the flexibility of all residues below 0.2 nm; (E) and (F) plots of time-dependent evaluation of the secondary structure changes of meuNa10 during the simulation, indicating that all secondary structure types were stable during simulation.

Tertiary structure of the meuNa10 obtained from three servers, along with the calculated Z-scores for all predicted models are illustrated in Figure 4. Z-score values of -6.47, -6.6, and -6.61 were measured for the models obtained from PHYRE2, I-TASSER, and SWISS-MODEL servers, respectively. All the Z-scores were within the acceptable area (-10 to 10), shown in Figure 4. However, the structure modeled by SWISS-MODEL server had the high quality, considering the more negative Z-score (Fig. 4C). In all three servers, homology modeling was performed based on anti-insect neurotoxin, LqhIT2 from *Leiurus quinquestriatus *(PDB code: 2I61) as a template. The structure of the meuNa10 is composed of a CS-alpha/beta domain, and an alpha-helix connecting to a three-stranded beta sheet utilizing eight cysteine residues (Fig. 4C). CS-alpha/beta domain, is a common structural motif in some scorpion peptides and proteins. To precisely compare the three models, we performed a structural alignment for the models obtained from different servers (Fig. 5). According to the structural alignment, the three models were very similar. RMSD, RMSF, Rg, and SASA were calculated by the analysis of trajectories resulting from MD simulation (Fig. 6). Based on the RMSD plot, the structure of meuNa10 had a steady state from 20 to 60 ns. After a fluctuation between 60 and 80 ns, it reached a steady state again (Fig. 6A). Overall, considering the RMSD value less than 0.5 nm for meuNa10, its structure showed low conformational changes during the simulation (Fig. 6A). RMSF was computed to determine the fluctuated residues of the meuNa10 during the MD simulation. As depicted in the RMSF plot (Fig. 6D), all residues had RMSF values less than 0.2 nm, indicating minor changes in meuNa10 structure. Furthermore, Rg plot (Fig. 6B) represented stability and compactness of the meuNa10 between 20 to 60 ns and after 80 ns. Analysis of the maintenance of the secondary structure using the DSSP program exhibited relative stability of the secondary structure of the meuNa10 model during the MD simulation (Fig. 6E and 6F). 

## DISCUSSION

Resistance to conventional insecticides and pesticides is widespread. Hence, finding alternative compounds with less side effects is a major concern. Protein-based anti-insect components from venomous animals are great candidates for designing and developing new bioinsecticides. Herein, we identified and characterized a putative anti-insect protein (meuNa10) in the transcriptome of the *M. eupeus* venom gland. 

Protein solubility is important to protein chemists, the pharmaceutical industry, and all biologists who work with protein in solution. Chemical and pharmaceutical application of proteins require a very high concentration of protein samples^[^^20^^]^. Different water solubility has been found for proteins. Crambin has been reported as a completely water-insoluble^[^^21^^]^, whereas the solubility more than 500 mg/mL has been determined for serum albumins^[^^22^^]^. Characterization of the meuNa10 has revealed that it is a water-soluble protein. Working with water soluble proteins creates some challenges, i.e., poor solubility of the proteins prevents the production of many industrial and useful therapeutic proteins^[^^23^^]^. Considering the high-water solubility identified for the meuNa10 herein, such above-mentioned problems will not be expected when working with this protein.

Literature reviews show that the negative values of GRAVY for a protein indicates its hydrophilicity^[^^24^^]^. Accordingly, meuNa10 with a GRAVY value of -0.69 is a hydrophilic protein. It has previously been revealed that for the rapid inactivation of the sodium channels, a hydrophobic protein is required^[^^25^^]^. Given the hydrophilic features predicted for meuNa10, it requires to increase the efficiency of meuNa10 to modulate the sodium channels by creating hydrophobicity-enhancing changes in amino acids. Previous studies have indicated that direct use of natural proteins as bioinsecticides is actually impossible. Multiple modifications on the newly found bioinsecticides are needed to provide an appropriate application in insect control. Increase in hydrophobicity, resistance to peptidase, being selective for organ action, and having agonistic or antagonistic activity are some important required changes^[^^26^^]^. Some insect proteins such as kinins^[^^27^^]^, proctolin^[^^28^^]^, sulphakinins^[^^29^^]^, myo-suppressins^[^^30^^]^, allatostatins^[^^31^^]^, and tachykinins^[^^32^^]^ are analogues of different natural proteins synthesized after some modifications,which make them suitable for using as insecticides.

The aliphatic index of neurotoxins originated from the scorpion was found to be 30.33 to 54.26^[^^24^^]^. Similarly, we reported an aliphatic index of 52.32 for the meuNa10. Since the origin of meuNa10 is a scorpion, the predicted value of aliphatic index for the meuNa10 seems reasonable. Notably, the instability index measures the stability of proteins in the experimental conditions. In this study, the instability index was less than 40, indicating that the protein is stable^[^^33^^]^. The value of the instability index obtained for the meuNa10 was 34.1, which verifies that the meuNa10 is a stable protein. However, a recent study comparing the instability index of proteins in vivo and in vitro, has stated that protein stability depends on not only its intrinsic nature but also its surrounding conditions. Therefore, determination of the instability index alone may not be a definitive indication for the stability of a protein^[^^34^^]^. However, it is suggested that this index can give a preliminary view of the target protein. Hence, further experimental research is needed to confirm the stability of meuNa10.

The Toxin_3 family is a domain found in the scorpion toxins and plant defensins^[^^35^^,^^36^^]^. It has already been established that the scorpion toxins containing Toxin_3 family target sodium channels and inhibit the activation of these channels. AaHIT, a toxin identified in the venom gland of scorpion *Androctonus australis*, which affects the insect’s NavS is composed of the domain Toxin_3 family^[^^37^^]^. Since meuNa10 contains a Toxin_3 family domain (Fig. 3), it likely has the sodium channel inhibitory activity.

The tertiary structure of meuNa10 represents the conserved CS-alpha/beta domain consisting of one alpha-helix and a three-stranded beta sheet, which are held together by three disulfide bridges (Fig. 4C). CS-alpha/beta domain is commonly found in scorpion peptides and proteins^[38]^. As the meuNa10 originates from scorpion, the presence of such a conserved motif can be justified. MD simulation results verify the predicted structure for meuNa10. On the other hand, beta finger-like strands of meuNa10, which are clearly visible in Figure 4C, can classify this protein in 3FP superfamily^[39]^. The 3FP superfamily contains small proteins, which typically consists of 60 to 80 amino acid residues. The protein members of this superfamily have a common tertiary structure, including three long finger-like beta strands that stabilized by disulfide bonds^[^^39^^]^. The proteins belong to the 3FP superfamily are nonenzymatic and have been well identified and explained in the venom gland of snakes^[^^40^^]^. However, a significant number of proteins have not yet been introduced in the scorpions. The meuNa10 identified in this study is one of the first 3FP proteins reported in the scorpions.

The present analysis of the transcriptome of the *M. eupeus* venom gland led to the identification of meuNa10 protein. Calculation of the physicochemical properties, as well as the structural analysis of this protein, revealed that meuNa10 is a potent, stable and water-soluble protein with a conserved motif in its structure with the ability to affect the function of insect sodium channels. Accordingly, it is an appropriate candidate for producing new bioinsecticides. However, more in vivo and in vitro studies are needed to approve the function of this protein and more investigate the activity of meuNa10. 

## DECLARATIONS

### Acknowledgments

The authors gratefully thank the Ahvaz Jundishapur University of Medical Sciences, Ahvaz, Iran for the financial support of this study.

### Ethical statement

Ethics approval was obtained from the research Ethics Committee of the Ahvaz Jundishapur University of Medical Sciences, Ahvaz, Iran (ethical code: IR.AJUMS.REC.1400.265).

### Data availability

Data supporting this article are included within the article.

### Author contributions

MB: conceptualization, running MD simulation, analysis of the results, and editing the final draft; MM: bioinformatics analysis, and editing the draft; MNS: preparation of the transcriptome and writing first draft; SJ: final analysis and final revising the manuscript. 

### Conflict of interest

None declared.

### Funding/support

This project was financially supported by Ahvaz Jundishapur University of Medical Sciences, Ahvaz, Iran (grant number: TRC0023).

## References

[1] Suhas R (2022). Structure, function and mechanistic aspects of scorpion venom peptides-A boon for the development of novel therapeutics. European journal of medicinal chemistry reports.

[2] Díaz García A, Varela D (2020). Voltage-Gated K+/Na+ channels and scorpion venom toxins in cancer. Frontiers in pharmacology.

[3] Quintero Hernández V, Jiménez Vargas JM, Gurrola GB, Valdivia HH, Possani LD (2013). Scorpion venom components that affect ion-channels function. Toxicon.

[4] Romanova DY, Balaban PM, Nikitin ES (2022). Sodium channels involved in the initiation of action potentials in invertebrate and mammalian neurons. Biophysica.

[5] Gamal El Din TM, Lenaeus MJ (2022). Fenestropathy of voltage-gated sodium channels. Front pharmacol.

[6] Dong K (2007). Insect sodium channels and insecticide resistance. Invertebrate neuroscience.

[7] Zhorov BS, Dong K (2017). Elucidation of pyrethroid and DDT receptor sites in the voltage-gated sodium channel. Neurotoxicology.

[8] Field LM, Emyr Davies TG, O'Reilly AO, Williamson MO, Wallace B A (2017). Voltage-gated sodium channels as targets for pyrethroid insecticides. European biophysics journal.

[9] Siegwart M, Graillot B, Blachere Lopez C, Besse S, Bardin M, Nicot PC, Lopez Ferber M (2015). Resistance to bio-insecticides or how to enhance their sustainability: a review. Frontiers in plant science.

[10] Oliveira AS, Fantinel AL, Artuzo FD, Oliveira L, Singer RB, da Frota Júnior MLC, Dewes H, Talamini E (2021). Applications of venom biodiversity in agriculture. EFB bioeconomy journal.

[11] Brown GB, Gaupp JE, Olsen RW (1988). Pyrethroid insecticides: stereospecific allosteric interaction with the batrachotoxinin-A benzoate binding site of mammalian voltage-sensitive sodium channels. Molecular pharmacology.

[12] Lombet A, Mourre C, Lazdunski M (1988). Interaction of insecticides of the pyrethroid family with specific binding sites on the voltage-dependent sodium channel from mammalian brain. Brain research.

[13] Ortiz E, Possani LD (2015). The unfulfilled promises of scorpion insectotoxins. The journal of venomous animals and toxins including tropical diseases.

[14] Naderi Soorki M, Jalali A, Baradaran M (2017). Improved system for constructing bacterial cDNA libraries from the venom glands of two iranian scorpions. Jundishapur university of medical sciences.

[15] Tamura K, Stecher G (2021). Kumar S. MEGA11: Molecular evolutionary genetics analysis version 11.Molecular biology and evolution.

[16] Wiederstein M, Sippl MJ (2007). ProSA-web: interactive web service for the recognition of errors in three-dimensional structures of proteins. Nucleic acids research.

[17] Gupta CL, Akhtar S, Bajpaib P, Kandpal KN, Desai GS, Tiwari AK (2013). Computational modeling and validation studies of 3-D structure of neuraminidase protein of H1N1 influenza A virus and subsequent in silico elucidation of piceid analogues as its potent inhibitors. EXCLI journal.

[18] Abraham MJ, Murtolad T, Schulzb R, Pall S, Smit J, Hessa B, Lindahl E (2015). GROMACS: High performance molecular simulations through multi-level parallelism from laptops to supercomputers. SoftwareX.

[19] Mafakher L, Rismani E, Rahimi A, Enayatkhani M, Azadmanesh K, Teimoori-Toolabi L (2022). Computational design of antagonist peptides based on the structure of secreted frizzled-related protein-1 (SFRP1) aiming to inhibit Wnt signaling pathway. Journal of biomolecular structure and dynamics.

[20] Kramer RM, Shende VR, Motl N, Pace CN, Scholtz JM (2012). Toward a molecular understanding of protein solubility: increased negative surface charge correlates with increased solubility. Biophysical journal.

[21] Ahn HC, Juranić N, Macura S, Markley JL (2006). Three-dimensional structure of the water-insoluble protein crambin in dodecylphosphocholine micelles and its minimal solvent-exposed surface. Journal of the American chemical society.

[22] Hutapea TPH, Madurani KA, Syahputra MY, Hudha MN, Asriana AN, Suprapto (2023). Albumin: Source, preparation, determination, applications, and prospects. Journal of Science: Advanced Materials and Devices.

[23] Hon J, Marusiak M, Martinek T, Kunka A, Zendulka J, Bednar D (2021). SoluProt: prediction of soluble protein expression in Escherichia coli. Bioinformatics.

[24] Panda S, Chandra G (2012). Physicochemical characterization and functional analysis of some snake venom toxin proteins and related non-toxin proteins of other chordates. Bioinformation.

[25] West JW, Patton DE, Scheuer T, Wang Y, Goldin AL, Catterall WA (1992). A cluster of hydrophobic amino acid residues required for fast Na(+)-channel inactivation. Proceedings of the national academy of sciences of the United States of America.

[26] Nowicki P, Kuczer M, Schroeder G, Czarniewska E (2021). Disruption of insect immunity using analogs of the pleiotropic insect peptide hormone Neb-colloostatin: a nanotech approach for pest control II. Scientific reports.

[27] Nachman RJ, Isaac R E, Coast G M, Holman G M (1997). Aib-Containing analogues of the insect kinin neuropeptide family demonstrate resistance to an insect angiotensin-converting enzyme and potent diuretic. Peptides.

[28] Kuczer M, Szeszel Fedorowicz W, Rosińskiand G, Konopińska D (1998). New proctolin analogues: Synthesis and biological investigation in insects. Letters in peptide science.

[29] Yu N, Benzi V, João Zotti M, Staljanssens D, Kaczmarek K, Zabrocki J, Nachman RJ, Smagghe G (2013). Analogs of sulfakinin-related peptides demonstrate reduction in food intake in the red flour beetle, Tribolium castaneum, while putative antagonists increase consumption. Peptides.

[30] Starratt AN, Lange AB, Orchard I (2000). Nterminal modified analogs of HVFLRFamide with inhibitory activity on the locust oviduct. Peptides.

[31] Xie Y, Peng Kai Z, Tobe SS, Deng XL, Ling Y, Qin Wu X, Huang J, Zhang L, Ling Yang X (2011). Design, synthesis and biological activity of peptidomimetic analogs of insect allatostatins. Peptides.

[32] Nachman RJ, Muren JE, Isaac RE, Lundquist CT, Karlsson A, Nässel DR (1998). An aminoisobutyric acid-containing analogue of the cockroach tachykinin-related peptide, LemTRP-1, with potent bioactivity and resistance to an insect angiotensin-converting enzyme. Regulatory peptides.

[33] Guruprasad K, Reddy BV, Pandit MW (1990). Correlation between stability of a protein and its dipeptide composition: a novel approach for predicting in vivo stability of a protein from its primary sequence. Protein engineering.

[34] Gamage DG, Gunaratne A, Periyannan GR, Russell TG (2019). Applicability of instability index for in vitro protein stability prediction. Protein and peptide letters.

[35] Assadi Porter FM, Aceti DJ, Markley JL (2000). Sweetness determinant sites of brazzein, a small, heat-stable, sweet-tasting protein. Archives of biochemistry and biophysics.

[36] Ceci LR, Volpicella M, Rahbé Y, Gallerani R, Beekwilder J, Jongsma MA (2003). Selection by phage display of a variant mustard trypsin inhibitor toxic against aphids. The Plant journal.

[37] Loret EP, Martin-Eauclaire M F, Mansuelle P, Sampieri F, Granie C, Rochat H (1991). An anti-insect toxin purified from the scorpion Androctonus australis Hector also acts on the alpha- and beta-sites of the mammalian sodium channel: sequence and circular dichroism study. Biochemistry.

[38] Tarr DE (2016). Establishing a reference array for the CS-αβ superfamily of defensive peptides. BMC research notes.

[39] Kini RM, Doley R (2010). Structure, function and evolution of three-finger toxins: Mini proteins with multiple targets. Toxicon.

[40] Kini RM, Koh CY (2020). Snake venom three-finger toxins and their potential in drug development targeting cardiovascular diseases. Biochemical pharmacology.

